# Impact of air recirculation and humidification systems on wood dust exposure during woodworking

**DOI:** 10.1093/annweh/wxaf027

**Published:** 2025-06-02

**Authors:** Anne Straumfors, Ine Pedersen, Erika Zardin Brinchmann, Torunn Kringlen Ervik, Anani Afanou, Kristine H Anmarkrud, Monica Eidhammer, Oda A H Foss, Nils Petter Skaugset

**Affiliations:** Occupational Toxicology Group, National Institute of Occupational Health, P.O. box 5330 Majorstuen, Oslo, Norway; Group of Chemical Work Environment, National Institute of Occupational Health, P.O. box 5330 Majorstuen, Oslo, Norway; Group of Chemical Work Environment, National Institute of Occupational Health, P.O. box 5330 Majorstuen, Oslo, Norway; Group of Chemical Work Environment, National Institute of Occupational Health, P.O. box 5330 Majorstuen, Oslo, Norway; Occupational Toxicology Group, National Institute of Occupational Health, P.O. box 5330 Majorstuen, Oslo, Norway; Occupational Toxicology Group, National Institute of Occupational Health, P.O. box 5330 Majorstuen, Oslo, Norway; Group of Chemical Work Environment, National Institute of Occupational Health, P.O. box 5330 Majorstuen, Oslo, Norway; Occupational Toxicology Group, National Institute of Occupational Health, P.O. box 5330 Majorstuen, Oslo, Norway; Group of Chemical Work Environment, National Institute of Occupational Health, P.O. box 5330 Majorstuen, Oslo, Norway

**Keywords:** softwood dust, hardwood dust, endotoxins, monoterpenes, resin acids, microbial exposure

## Abstract

Employees in the woodworking industry, including carpentry workshops, wood product factories, and the wooden house industry, are exposed to wood dust at work. In Norway, this industry is exempt from regulations banning air recirculation, intended to prevent harmful substance buildup in working environments. While wood dust exposure is linked to increased risks of cancer and respiratory diseases, eliminating the exemption could have significant economic consequences for companies reliant on heated air recirculation during winter. A detailed characterization of the exposure is needed to evaluate the health risks associated with recirculated air. Wood dust contains components like resin acids, endotoxins, fungi, bacteria, monoterpenes, and aldehydes, which can irritate the skin, eyes, and respiratory system. Understanding these exposures is crucial for evaluating whether existing occupational exposure limits (OELs) adequately protect workers’ health. This study aimed to assess wood dust and associated exposures in companies with and without air recirculation or humidification. Between 2019 and 2023, full-shift personal aerosol sampling was conducted in 23 companies during winter. Samples were analyzed for wood dust mass, endotoxin, bacteria and fungi, resin acid, monoterpenes, and aldehydes. Log-transformed exposure data were analyzed by mixed models using company types and work-related conditions as fixed effects. Results showed average exposure below OELs but with significant variability. About 25% of measurements exceeded the OEL for inhalable wood dust of 1 mg/m^3^. Air recirculation had mixed effects; it lowered the monoterpene exposure by 95% (from GM 597 µg/m^3^ to GM 27 µg/m^3^) but increased the GM microbial exposure 2 to 5 times across companies. The impact of air recirculation varied across company types. For building element production, it nearly doubled the wood dust exposure from soft woods (from GM 0.15 mg/m^3^ to GM 0.27 mg/m^3^), while for door/window manufacturers, exposure was nearly halved compared to those not using air recirculation (from GM 0.44 mg/m^3^ to GM 0.25 mg/m^3^). Air humidification lowered the inhalable dust exposure by 59% across the company (from GM 1.36 mg/m^3^ to 0.56 mg/m^3^) but led to increases in monoterpene by 90 % (from GM 86 µg/m^3^ to GM 792 µg/m^3^) and microbial exposure by up to 64%. Companies manufacturing interior products without a humidification system had resin acid exposure levels that were 10 times higher (GM 3323 ng/m^3^) compared to those with a humidification system (GM 344 ng/m^3^). The variability in exposures was mostly influenced by company-specific practices. Evaluation of preventive measures should therefore be tailored to the individual company.

**What’s Important About This Paper?**

The woodworking industry is exempt from regulations banning air recirculation, which are intended to prevent harmful substance buildup, in working environments. This study found that air recirculation, air humidification, and production types had varied and complex impacts on workers’ exposure to wood dust, resin acids, and microbial and volatile components in the woodworking industry. The findings enhance the understanding of exposure levels in relation to the current regulations and provide an improved basis for assessing whether the existing legislation (with exemptions) offers sufficient protection for workers in the woodworking industry.

## Introduction

Workers in carpentry, wood products manufacturing, and the wooden house industry are regularly exposed to wood dust during woodworking (Brosseau et al. 2001; Kauppinen et al. 2006). Exposure to wood dust is associated with an increased risk of nasal and sinus cancer (IARC 2012), with highly exposed workers also facing a higher risk of lung cancer. Other respiratory effects have also been reported at exposure levels lower than those typically associated with cancer risk (Demers et al. 1995). Studies from sawmills and the pulp and paper industry have shown that exposure to wood dust is linked to respiratory effects and diseases, such as asthma, chronic bronchitis, rhino-conjunctivitis, and acute and chronic lung function changes (Shamssain 1992; Talini et al. 1998; Bohadana et al. 2000; Douwes et al. 2001; Borm et al. 2002; Schlünssen et al. 2002a, b; Skovsted et al. 2003; Schlünssen et al. 2004; Jacobsen et al. 2008).

The exposure–response relationship in epidemiological studies is complex and requires further examination of wood dust’s chemical and biological components. Wood, primarily composed of cellulose, polyose, and lignin, also contains volatile compounds like monoterpenes, which vary by species and can cause eye, throat and respiratory irritation, chest tightness, pulmonary impairment, increased bronchial activity, and airway inflammation (Hedenstierna et al. 1983; Johard et al. 1993; Dahlqvist and Ulfvarson 1994; Eriksson et al. 1996). Other volatile organic compounds (VOCs) from wood or wood-based materials such as plywood, chipboard, fiberboard, and other boards containing adhesives can further contribute to health problems (Skulberg et al. 2019) (Rohr 2013; Pytel et al. 2022). Exposure to formaldehyde in the production and use of particle board and glued wood has been linked to cancer (IARC 1995) and irritations of the eyes and skin, respiratory ailments, and inflammation of the nose. The wood contains naturally occurring resin that can vary between tree species. Abietic acid is one of the main resin acids in spruce and pine and has been associated with allergic sensitization, respiratory symptoms, and asthma when working with pine (Ayars et al. 1989; Hessel et al. 1995; Demers et al. 1997). Low levels of endotoxins and microorganisms have previously been found in the furniture industry (Alwis et al. 1999; Jacobsen et al. 2010; Gioffrè et al. 2012). However, it is not known whether conditions for any microbiological growth may occur in situations related to air recirculation or ventilation stops. Inhalable exposure to airborne spores is known to cause allergic alveolitis among employees at sawmills, while endotoxins, highly inflammatory cell wall components of Gram-negative bacteria, have been associated with the development of respiratory ailments in a variety of industries (Liebers et al. 2020)

The prevalence of wood dust exposure in the workforce varies between 1.4% and 5.5% (Olsson and Kromhout 2021). In the EU, 3.6 million workers, or 2% of the total workforce, are exposed to inhalable wood dust (Kauppinen et al. 2006). Regulations vary, with Norway setting the 8 h time-weighted average occupational exposure limit (OEL) for softwood (Nordic species except oak and beech) dust to 2 mg/m^3^ and hardwood (tropical species, oak and beech) dust at 1 mg/m^3^ inhalable dust (Norwegian Labour Inspection Authority 2024) while France enforces a universal limit of 1 mg/m^3^ (Garras et al. 2023). Other regions set limits between 1-5 mg/m^3^, depending on the wood type and country's standards (European Parliament 2017) (HSE 2020; Occupational Safety and Health Administration (OSHA) 2023).

Workplace conditions like ventilation, humidification, and dust extraction systems affect exposure levels. Recirculated air systems, used for heat and moisture retention in colder months, can elevate the risk of exposure to harmful substances (Eriksson et al. 1997; Schlünssen et al. 2008; Hagström et al. 2012). Several countries which have a general workplace prohibition on air recirculation to avoid harmful buildup, also exempt the woodworking industry from this for practical and economic reasons, providing that exposure levels are within the regulated limits (Ministry of Labour and Social Inclusion 2023; Danish Working Environment Authority 2024; Swedish Work Environment Authority 2023). This highlights the need for consistent exposure monitoring to ensure worker safety. So far, no documentation exists of whether today’s practice with air recirculation in the woodworking industry sufficiently addresses indoor air quality and occupational health related to exposure to wood dust and associated compounds. A systematic exposure characterization is important for obtaining more knowledge of the exposure levels associated with the current woodworking practice.

This study aims to (i) assess wood dust exposure levels across the woodworking industry using standardized sampling and evaluation against OELs; (ii) survey exposure to related components, including resin acids, terpenes, aldehydes, and microbial components; and iii) evaluate potential exposure differences between facilities with and without air recirculation or humidification.

## Materials and methods

### Study design and study population

Measurements were conducted during winter seasons to compare exposures between companies using air recirculation during the heating season and those that did not. Winter was chosen for better control of ventilation conditions due to reduced natural ventilation. Member companies of Norwegian Wood Products (Norske Trevarer) and Housing Producers’ Association (Boligprodusentenes Forening) participated in the program. A total of 324 full-shift wood dust samples were collected from 128 workers across 23 companies during the winters of 2019 to 2023. Additionally, 41 to 98 samples of wood-dust-related components were collected at six companies. Thirteen companies used air recirculation, nine did not, and one provided no information about this. Sixteen companies worked only with softwood, while seven used both softwood and hardwood. None of the companies worked only with hardwood. Participating companies represented various wood products manufacturers and tasks (Table 1). Information on ventilation, air recirculation, and other exposure-related conditions was collected from a questionnaire completed by companies or administered during sampling. Humidification systems were fixed (automatically maintaining 20% to 50% relative humidity), ceiling-mounted mist systems, or a combination of both.

**Table 1.** Types of participating companies

Industry codes (NACE)	Production type	Tasks	Number of companies	Number of workers	Number of samples
16.232	Stairs	CNC milling machine, sawing, sanding, cutting, grinding, varnishing, oiling, insert bearing, milling MDF, splitting	3	15	42
16.232 31.090 31.020	Interior products (benches/counters, shelving and kitchen fittings, wardrobes)	Milling, sawing, sanding, CNC, varnishing, planing, production and assembling work, carpentry, MDF, oiling, glueing, splitting	4	24	51
16.231 16.232	Building elements (wall elements, floor elements, ceiling elements, roof trusses, garage doors)	Construction, production and assembling work, making holes, insulating, cutting, assembly, nailing, pressing, woodworking, sawing, blowing, splitting, fitting, sanding, milling	11	60	149
16.232	Door/window frames	Milling, drilling, woodworking, rebating, CNC, planing, cutting, sawing, machining frame parts, molding, razing, sanding, grinding, joining	5	29	82
Total number of companies, workers and samples			23	128	324

CNC: computer numerical control; MDF: medium density fiberboard.

### Sampling and analytical methods

#### Personal sampling with repeated measurements

Active samplers were placed in the workers’ breathing zone at the start of their work shift and were retrieved at the end. Workers in the six companies where multiple components were sampled, carried five samplers powered by four pumps, all housed in a backpack. One pump with dual tube and individual flow adjustment was used for the simultaneous monoterpenes and aldehyde sampling, one for endotoxins, one for resin acids, and one for wood dust (inhalable or total). Pumps were calibrated to the specific flow rates for each sampler, and the average of start and end flow rates (L/min) and sampling time (min) determined the collected air volume. Mean, minimum, and maximum sampling time and volume for each sampler type are detailed in Table S1. Each worker underwent one to seven repeated measurements.

#### Sampling of airborne wood dust

The total dust fraction was collected on 25 mm PVC filters (5 μm pore size, Merck KGaA, Darmstadt, Germany) in black antistatic total dust samplers (Pall Laboratories, Port Washington, NY, USA) (2 L/min) at companies working with Nordic soft woods, except oak and beech. One company used 3 μm Teflon filters, assumed to have a comparable efficiency. An antistatic intermediate piece minimalized wall deposition. The inhalable fraction was collected on 37 mm PVC filters (5 μm pore size, Merck KGaA,) in CIS samplers (Conical Inhalable Sampler, J S Holdings, Hertfordshire, UK) (3.5 L/min) at companies handling hard, exotic woods, oak, beech, or mixed woods.

#### Gravimetric determination of dust mass

Dust masses were determined gravimetrically. At STAMI, filters were weighed using a micro scale (Sartorius MC-5, Göttingen, Germany) in a climate-controlled room (20±1 °C, 40±2%) after acclimatization for at least one day. Field blanks were analyzed for every ten samples, and any mass change was used for adjustment. Quality control with reference weights and filters was conducted before each weighing sequence. Static charge was removed with a Po^210^ source (Staticmaster®, NRD, LLC, NY, USA). The gravimetric limit of quantification (LOQ) was 0.02 mg/filter. Weighing procedures for companies that used other laboratories than STAMI, are not provided.

#### Measurement of resin acids

The resin acids in the inhalable fraction were collected on 37 mm PVC filters (5 μm pore size, Merck KGaA) in CIS samplers (3.5 L/min). Samples were prepared as previously described (Axelsson et al. 2011). In brief, filters were frozen in 4 mL glass vials until extraction with methanol and internal standard. Prior to analysis samples were filtered using a Millex-GN 0.20 um 13 mm nylon syringe filters (Merck KGaA). Analysis with liquid chromatography with mass spectrometric detection (LC-MS) (Thermo Scientific Dionex Ultimate 3000 RSLC UHPLC coupled with a Thermo Scientific TSQ Vantage EMR triple quadrupole) was performed using a 150 × 2.1 mm ACE Excel 3 C18-amide column (Teknolab) with a pre-column filter (Ace UHPLC), APCI ion source and isocratic mobile phase of acetonitrile and 30 mM ammonium acetate pH 6.3 (70:30, v/v) with a flow rate of 0.3 mL/min (Latorre et al., 2003). The resin acids 7-oxodehydroabietic acid (7-OXO), dehydroabietic acid (DHAA), levopimaric acid (LPA), abietic acid (AA), and isopimaric acid (IPA) were determined with an LOQ of 50 ng. Exposure levels are presented as ng/m^3^ for each component individually (Table S2), and the sum of all resin acids are used in the subsequent data analyses. All individual resin acids were correlated (*r*_p_ = 0.47 to 0.95, *P* < 0.0001).

## Volatile compounds

### Monoterpenes

Monoterpenes were collected on charcoal tubes (Anasorb CSC 226-01, Skcltd.com) with 0.05 L/min flow (NIOSH, 1996, 2003) and desorbed overnight in 3 mL carbon disulfide. α-pinene, β-pinene, d-limonene and 3-karene were quantified using an Agilent 7890 gas chromatograph (Agilent Technologies, Santa Clara, CA, USA) with an Agilent HP-5 (0.32 mm × 25 m, 1.05 µm film thickness) capillary column and flame ionization detector and an Agilent 6890 GC-FID with an Agilent CP-TCEP (50 m × 0,25 mm, 0,40 µm film thickness) capillary column. The LOQs were 0.004 ppm (β-pinene) and 0.003 ppm (all other). Exposure levels are presented as ng/m^3^ for each component individually (Table S1), and the sum of all monoterpenes are used in the subsequent data analyses. All individual monoterpenes were correlated (*r*_p_ = 0.87 to 0.94, *P* < 0.0001).

### Aldehydes

Aldehydes (formaldehyde, acetaldehyde, acrolein) were collected on DNPH-impregnated Sep-Pak cartridges (WAT037500, Waters.com) with 0.05 L/min flow (NIOSH 2016) eluted with 3 mL acetonitrile (Rathburn Chemicals Ltd, Walkerburn, Scotland), filtered using a 3 mL BD syringe (BD Biosciences, Art. No. 309657) with attached Acrodisc syringe filter (13 mm with 0.2 µm PVDF Membrane, Pall Corporation) and quantified using an Agilent 1100 Series liquid chromatograph (Agilent Technologies) with a Kromasil C_18_ column (4.5 mm × 150 mm, pore size 100 Å and particle size 3.5 µm, Nouryon, Amsterdam, Netherlands, Art. No. MH3CLA15), an ultraviolet detector and an isocratic mobile phase of acetonitrile and milliQ water (Merck KGaA) (50/50, v/v) at 1 mL/min. The LOQ ranged from 0.0014 to 0.0048 mg/m^3^ for formaldehyde and from 0.0019 to 0.0072 mg/m^3^ for acetaldehyde depending on the analytical series. Three and seven of the 96 samples, respectively (3% and 7%), were below the LOQ.

### Measurement of microbial components Quantification of endotoxin activity

Ninety-four samples were collected with a personal inhalable aerosol sampler (PAS-6) with a 25 mm fiberglass filter, type GF/A (Whatman, Merck KGaA), and 2 L/min flow. The filters were extracted with pyrogen-free water and the amount of biologically active endotoxin was determined with quantitative kinetic chromogenic Limulus Amoebocyte Lysate test (Douwes et al. 1995). Fourteen samples (15%) were below the LOQ of 0.05 EU/ml.

### Quantification of fungal spores

Total dust was collected on 25 mm polycarbonate (PC) filters (0.8 μm pore size, Merck KGaA) and transferred to centrifugation tubes with 5 mL phosphate buffer containing 0.1% bovine serum albumin. After 5 min of sonication and 60 min of vortexing, the filter was removed. Depending on the dust mass, 0.5 mL or 1 mL of suspension was filtered onto a 25-mm PC filter (0.4 μm pore size) for fungal and actinobacterial spore analysis using field-emission scanning electron microscopy (SU 6600 Hitachi, Ibaraki-Ken, Japan). Filters were air-dried, mounted on 25 mm aluminum stubs, platinum-coated (approx. 5 nm), and examined at 3000× magnification with spores counted in 100 random fields with area of 1064 μm^2^ each as previously described (Afanou et al. 2015). Spore counts were extrapolated to the entire filter and normalized to air volume. Of 41 samples from six companies, 39 (95%) had no spores. Determination limit was 10,600 or 21,300 spores per filter for 1 mL or 0.5 mL suspensions, respectively.

### DNA analysis of fungi and bacteria

Prevalence of bacteria and fungi in inhalable and total dust was investigated by detection of DNA from preserved regions of bacteria and fungi, respectively. Filter elution and DNA extraction were performed as previously described (Straumfors et al. 2019). The number of bacterial and fungal genomic copies present in the sample was analyzed with specific primers for 16S and 18S conserved DNA regions, respectively, in droplet digital polymerase chain reaction (ddPCR) (Eriksen et al. 2023) using a Bio-Rad QX200 droplet generator and a Bio-Rad QX200 droplet reader (Bio-Rad Laboratories Inc., CA, USA).

### Data analysis

STATA/SE 15.1 software (StataCorp LP, Collage Station TX, USA) was used for statistical analysis. Positive values below LOQ for total dust, resin acids, endotoxin, monoterpenes, and aldehydes were used as they were, whereas zero values were replaced with LOQ/ $\sqrt[{}]{2}$ and then divided by the individual sampling volume, in order to include these samples in the data analyses (Succop et al. 2004; Ogden 2010). As the exposure data were skewed and approximated a log-normal distribution, the values were ln-transformed. The exposure concentrations for each component are given as arithmetic mean (AM) and AM standard deviation (SD), median with minimum and maximum, geometric mean (GM) of the observed values and 95% confidence interval (CI), and GM adjusted for random effects (GM_adj_) and 95% CI. To assess the effect of companies, production types, air recirculation, air humidification, and other determinants of relevance to exposure, we performed mixed model regression analyses using determinants as fixed effect variables and the identity of workers as random effect. The assumptions of normal distribution, independence, and constant variance of the residuals, as well as linear relationship between explanatory variables and the exposures were met. Independence was considered by including the random intercept in the mixed model, and possible time dependence in the residuals due to repeated sampling of the same persons were excluded by confirming that the inclusion of an autoregressive term for the residuals had no effect. The assumption of constant variance of the residuals were checked visually by plotting residuals against fitted values. The variance inflation factor was used to check possible collinearity between fixed effect variables of the same model. Interactions between production types and other determinants were also tested. The influence of adding fixed effect variables and interaction terms into the models was tested by likelihood ratio test using the maximum likelihood function with a *P*-level ≤0.05 considered statistically significant. A general expression of the resulting models can be presented as:

y = µ+β + u +ɛ

where y is the predicted exposure, µ is the constant/intercept, β is the regression coefficient of the fixed effects, u is the random intercept for worker (the between worker (bw) variance (δ^2^bw)), and ɛ is the residual term (within worker (ww) variance (δ^2^ww)).

The estimated geometric mean exposure and 95% CI was calculated from the mixed models by the margins postestimation command in STATA. To quantify the contribution of the fixed effects to the bw and ww variance components, values of the variance components obtained from the mixed models were compared with those from a pure random effects model. The percentage of the variances that could be explained by the fixed effect variables was calculated as follows: (var_random_-var_mixed_)/var_random_ × 100%. Models used as basis for estimation of exposure and explained variance are detailed in the Tables S4–S8.

## Results

### Exposure characterization

The overall results of the personal measurements are shown in Table 2. The average exposure was low for all components compared with the Norwegian OEL for wood dust of 1 mg/m^3^ inhalable dust from hard woods and 2 mg/m^3^ total dust from soft woods, 140 mg/m^3^ for monoterpenes, 0.37 mg/m^3^ for formaldehyde and 45 mg/m^3^ for acetaldehyde (Norwegian Labour Inspection Authority 2024). The microbial exposure was also low compared to the proposed limit values for endotoxin of 90 EU/m^3^ (Health Council of the Netherlands 2010) and for general spores of 10^5^ spores/m^3^(Eduard 2009). Fungal spores were observed in only two of the samples. There was, however, considerable variation between measurements. Some wood dust measurements were more than ten times higher than average, thus exceeding the limit values. Seven and 28% of the total dust and inhalable dust measurements, respectively, exceeded the respective OELs. Fifteen and nearly seven hundred times higher exposure than the median exposure of resin acids and monoterpenes, respectively, were observed. Inhalable AA exposure correlated weakly with inhalable dust, but the sum of resin acids did not correlate with inhalable dust exposure (Table S3). Some employees had 18 times higher exposure than average to endotoxins, and 260- and 46-times higher exposure to bacteria and fungi, respectively. Although there was considerable variation in the samples collected, the proposed limit values or observed effect levels were not exceeded in any of these cases.

**Table 2.** Exposure to wood dust, resin acids, volatile compounds, and microorganisms

Components	N	%<DL^1^	AM (SD)^2^	Median (min-max)	GM_obs_ (CI)^3^	GM_adj_ (CI)^4^
**Wood dust**						
Soft wood species (mg/m^3^)	242	0	0.50 (0.62)	0.30 (0.016–4.7)	0.30 (0.28–0.35)	0.29 (0.24–0.35)
Hard & soft wood species (mg/m^3^)	81	0	0.88 (1.09)	0.53 (0.07–6.1)	0.52 (0.42–0.65)	0.61 (0.45–0.82)
**Resin acids** (µg/m^3^)	98	1	2.19 (2.95)	1.24 (0.11–19.9)	1.12 (0.88–1.43)	1.10 (0.82–1.46)
**Volatile components**						
Monoterpenes (µg/m^3^)	77		6596 (14225)	79 (28–54095)	260 (149–458)	240 (115–501)
Formaldehyde (µg/m^3^)	97	3	29 (30)	19 (1–186)	17 (13–21)	17 (13–23)
Acetaldehyde (µg/m^3^)	97	7	2.0 (0.5)	1.8 (0.5–4.8)	1.9 (1.8–2.0)	1.9 (1.8–2.0)
**Microbial components**						
Endotoxin (EU/m^3^)	94	15	4.0 (5.9)	2.2 (0.017–39)	1.38 (0.93–2.05)	1.46 (0.01–2.35)
Fungal spores (spores/m^3^)	41	95	22,300 (10,300)	22,300 (0–32,700)	nc	nc
Bacteria (genomic copies/m^3^)	60	0	8,100 (13,600)	3,100 (280–815,500)	2,300 (2,400–4,800)	3400 (2100–5400)
Fungi (genomic copies/m^3^)	60	0	1,585 (4,100)	660 (0–30,500)	630 (450–890)	640 (430–950)

^1^%<DL: Percent of samples with values below the detection limit; ^2^AM (SD): arithmetic mean (standard deviation); ^3^GM_obs_ (CI): geometric mean of the observed values (95% confidence interval); ^4^GM_adj_ (CI): GM adjusted for random effects (95% CI). nc: not calculated, spores were observed in only two samples.

### Factors influencing exposure

#### Air recirculation and humidification

The use of air recirculation had no significant effect on the general exposure or on the marginal mean total or inhalable wood dust, or resin acid exposure level across all types of companies (Table 3). However, exposure to endotoxins and bacteria were 2- and 5-folds higher, respectively, when using air recirculation compared with no air recirculation (Table 3), whereas the exposure to monoterpenes was on average over 20 times lower with air recirculation than without (GM 27 µg/m^3^ versus 597 µg/m^3^). The use of air humidification had a significant effect on the inhalable dust level (*P* = 0.04), reducing the mean exposure by nearly 2.5 times to 0.56 mg/m^3^ compared with 1.36 mg/m^3^ when no air humidification was used (Table 3, Table S7). The exposure to endotoxin and bacteria, as well as monoterpenes were significantly higher when using air humidification compared to no air humidification (Table 3). The exposure to formaldehyde were also higher with air humidification than without, however, the measured levels were still low. An apparent interaction effect between air recirculation and air humidification (not shown) turned out to be due to differences between product types, which varied in the use of air recirculation and air humidification, respectively (see below).

**Table 3.** Mean exposure with and without air recirculation and humidification in the workplace

	Air recirculation			No air recirculation			Air humidification			No air humidification		
**Exposure components**	**n**	**GM**	**95% CI**	**n**	**GM**	**95% CI**	**n**	**GM**	**95% CI**	**n**	**GM**	**95% CI**
Total dust (mg/m^3^)	85	0.25	0.18–0.36	138	0.26	0.21–0.32	70	0.20	0.14–0.28	131	0.27	0.21–0.34
Inhalable dust (mg/m^3^)	81	0.61	0.45–0.82	0	n.a.	n.a.	65	**0.56**	**0.40**–**0.78**	10	**1.36**	**0.61**–**3.02**
Resin acids (ng/m^3^)	45	900	593–1365	53	1307	882–1937	55	1024	698–1501	43	1200	772–1865
Endotoxins (EU/m^3^)	45	**2.1**	**1.0**–**4.0**	49	**1.1**	**0.6**–**2.0**	54	**2.2**	**1.2**–**4.0**	40	**0.8**	**0.4—1.7**
Bacteria (gc/m^3^)	39	**5950**	**3750**–**9450**	21	**1190**	**630**–**2260**	25	**6780**	**3790**–**12140**	29	**1550**	**910**–**2660**
Fungi (gc/m^3^)	39	740	450–1210	20	480	240–950	25	820	440–1550	28	480	270–860
Monoterpenes (µg/m^3^)	33	**27**	**20**–**163**	50	**597**	**261**–**1366**	33	**792**	**304**–**2065**	44	**86**	**35**–**209**
Formaldehyde (µg/m^3^)	44	18	12–27	53	16	11–25	54	**27**	**19**–**37**	43	**9**	**6**–**14**
Acetaldehyde (µg/m^3^)	44	**1.8**	**1.7**–**1.9**	53	**2.1**	**1.9**–**2.2**	54	**1.8**	**1.7**–**2.0**	43	**2.0**	**1.9**–**2.2**

Bold font indicate group differences with p value <0.01 in mixed models of exposure components using air recirculation or humidification as fixed effects and workers as random effects; n.a.: not available, all samples were collected with air recirculation. Forty-one samples of total dust, 6 samples of inhalable dust, and 6 samples of bacteria and fungi, respectively, did not have any associated data on the use of air humidification.

#### Companies and product types

There were significant differences in total dust exposure between companies as well as between some of the type of products produced across companies (Tables S4–S5). Workers at companies producing doors and window frames had significantly lower exposure to total dust (mean 0.19 mg/m^3^, 95%CI 0.14 to 0.26) than workers at companies producing building elements (mean 0.34 mg/m^3^, 95%CI 0.28 to 0.42; *P* = 0.01) (Table 4). The mean exposure was highest among workers at companies producing interior products (GM 0.47 mg/m^3^), but the exposure was highly variable and the difference was not statistically significant. The mean exposure level was nevertheless below the OEL of 2 mg/m^3^.

**Table 4.** Estimated personal exposure to wood dust, microbial and volatile compounds during woodworking^1^.

	Building elements			Door/window frames			Interior products			Stairs			Group differences^2^
**Exposure components**	**n**	**GM**	**95% CI**	**n**	**GM**	**95% CI**	**n**	**GM**	**95% CI**	**n**	**GM**	**95% CI**	
Total dust (mg/m^3^)	148	0.34	0.28–0.42	76	0.19	0.14–0.26	17	0.47	0.24–0.92	0	-	-	a, d
Inhalable dust (mg/m^3^)	0	-	-	6	0.51	0.19–1.37	33	0.87	0.58–1.31	42	0.42	0.27–0.64	f
Resin acid (ng/m^3^)	33	884	559–1399	20	2512	1389–4545	27	750	460–1224	18	1226	656–2290	a, d
Endotoxin (EU/m^3^)	30	0.40	0.20–0.80	19	4.60	2.0–10.8	27	3.90	2.0–7.7	18	0.70	0.30–1.80	a, b, e, f
Bacteria (gc/m^3^)	21	1200	650–2200	25	6780	3880–11880	8	3000	1130–8010	6	8150	2850–23370	a, c
Fungi (gc/m^3^)	20	480	250–940	25	820	450–1510	8	470	160–1390	6	830	260–2660	
Monoterpenes (µg/m^3^)	34	98	78–124	16	27550	19600–38725	27	57	45–73	0	-	-	a, b, d
Formaldehyde (µg/m^3^)	33	6.9	5.6–8.6	20	71	54–94	27	31	25––40	17	6.5	4.8–8.8	a, b, d, e, f
Acetaldehyde (µg/m^3^)	33	2.1	2.0–2.2	20	2.0	1.8–2.2	27	1.8	1.6–1.9	17	1.8	1.7–2.0	a, b

^1^The number of companies of each type were for total/inhalable dust: building elements (*n* = 11/0), door/window frames (*n* = 4/1), interior products (*n* = 1/3), stairs (*n* = 0/3). For all other exposure components, the numbers of companies were building elements (*n* = 2), door/window frames (*n* = 1), interior products (*n* = 2), and stairs (*n* = 1). ^**2**^Significant group differences with a *P*-value ≤0.05 in pairwise comparisons in mixed models is marked as a) building elements vs door/window frames, b) building elements vs interior products, c) building elements vs stairs, d) door/window frames vs interior products, e) door/window frames vs stairs, f) interior products vs stairs; *n* = number of measurements; gc= genomic copies. Models are given in Table S4.

There were also significant differences in inhalable dust exposure between companies, whereas the differences between product types were not statistically significant (Tables S4–S5).

Differences in resin acid exposure were observed both between companies and between product types across companies (Tables S4–S5). Workers at the company producing door and window frames were significantly higher exposed to resin acids (mean 2512 ng/m^3^, 95%CI 1389 to 4545) compared with those producing building elements (mean 884 ng/m^3^, 95%CI 559 to 1399) or interior products (mean 750 ng/m^3^, 95%CI 460 to 1224) (Table 4).

Fungal spores were observed in only two samples, from two different company types (building elements and doors/windows, respectively), and were not included in further analyses. The exposure to other microbial components was generally low, however varied somewhat between companies and product types (Tables S4–S5, Table 4). The endotoxin exposure was higher among doors/windows producers (mean 4.6 EU/m^3^) as well as producers of interior products (mean 3.8 EU/m^3^) compared with producers of building elements (0.42 EU/m^3^) and stairs (0.72 EU/m^3^) (Table 4). The exposure to bacteria was highest among stairs producers (mean 8150 genomic copies/m^3^) and doors/windows producers (6780 gc/m^3^), whereas those who produced building elements and interior products were significantly lower exposed (*P* < 0.001 to 0.002, Table 4). The same trend could be seen for fungal exposure, although at even lower levels.

Exposure to volatile components (monoterpenes, formaldehyde, and acetaldehyde) was generally low but varied between companies (Tables S4–S5). Monoterpenes exposure was lower in companies manufacturing interior products and building elements compared to the one producing door and windows. Conversely, formaldehyde exposure was higher among door and window manufacturers than any other category. Despite this variation, the exposure levels remained low overall (Table 4).

A strong interactive effect between air recirculation and the production of doors/windows and building elements, respectively, was shown for total dust exposure when including product type and air recirculation in the same model (*P* < 0.01). This revealed that companies producing doors/windows using air recirculation had significantly lower exposure than the other types of producers and the ones not using air recirculation (0.15 mg/m^3^ vs 0.27 mg/m^3^) (Table 5, Table S6). It was the opposite for companies producing building elements, that had an exposure of 0.44 mg/m^3^ with air recirculation and 0.25 mg/m^3^ without air recirculation (Table 5).

**Table 5.** Total dust concentrations (mg/m^3^) by company type with or without air recirculation

	Air recirculation			No air recirculation		
Production	*n*	GM	95%CI	*n*	GM	95%CI
Door/windows	52	0.15	0.10–0.22	30	0.27	0.18–0.42
Interior products	51	0.47	0.25–0.89	0	n.e.	n.e.
Building elements	22	0.44	0.25–0.77	108	0.25	0.20–0.33

Geometric mean and 95%CI of total dust estimated by mixed models (Table S6). n.e.: not estimable due to no observation in interaction group.

Companies producing interior products without air humidification had 10 times higher resin acid exposure than similar companies with air humidification (*P* < 0.001) (Table 6).

**Table 6.** Resin acid concentrations (ng/m^3^) by company type with or without air humidification

	Air humidification			No air humidification		
Production	*n*	GM	95%CI	*n*	GM	95%CI
Door/windows	20	2512	1567–4028	0	n.e.	n.e.
Interior products	17	344	210–564	10	3323	1705–6479
Building elements	0	n.e.	n.e.	17	881	611–1270
Stairs	18	1225	745–2016	0	n.e.	n.e.

Geometric mean and 95%CI of resin acids estimated by mixed models (Table S8). n.e.: not estimable due to no observations.

### Exposure variance explained by potentially influencing factors

Differences in production types explained only 3 % of the variance in total dust exposure, with BW and WW contributing equally (48% and 52%). Including air recirculation and its interaction with product type (Table S9 model 3) increased explained variance by just 2 %. For inhalable dust, production type explained 7% of the variance (BW 58% and WW 42 %), but including air humidification reduced this to 4%, with equal BW and WW contributions (Table S9 model 4).

Differences between individual companies explained more variance than production types (Table S9 model 2): 18% for total dust and 21% for inhalable dust. For other exposure components, production type explained 9%, 53%, 29%, and 3% for resin acids, endotoxin, bacteria, and fungi, respectively, and 99%, 43%, and 0.2%, respectively, for monoterpenes, formaldehyde and acetaldehyde (Table S9 model 1). Variance explained by companies were 23%, 69%, 37%, and 24% for resin acids, endotoxin, bacteria, and fungi, respectively, and 99%, 43%, and 1%, respectively, for monoterpenes, formaldehyde, and acetaldehyde (Table S9 model 2). For all components, except inhalable dust, within-worker differences contributed more (49% to 73%) to total variance than between-worker differences (27% to 51%).

Information on other company variables relevant to exposure, such as ventilation, extraction, filter use, and cleaning routines, was too complex for meaningful statistical analyses. Samples distribution across these variables and company types is detailed in the supplementary material (Tables S10–S13).

## Discussion

This study characterized exposure levels in the Norwegian woodworking industry, revealing that while average exposures were below existing OELs, significant variation existed, with some workers exceeding the limits for total or inhalable wood dust. Exposure to microbial and volatile components also varied but low levels indicated that these exposures were not a major concern in this study. The use of air recirculation affected individual exposures at the various types of companies differently, reducing the monoterpene exposure, but increasing the microbial exposure. Air humidification reduced the inhalable dust exposure across company type but increased monoterpene and microbial exposure. Factors potentially influencing the exposures were in general closely adapted to the individual companies, making it difficult to isolate their effects. As a result, differences between individual companies explained most of the variability in exposure.

### Exposure levels

Our findings indicate somewhat lower dust levels compared to similar studies, where inhalable wood dust exposures often exceeded 1 mg/m^3^ (Mandryk et al. 1999; Kauppinen et al. 2006; Gioffrè et al. 2012; Simpson et al. 2024). For example, a study across 25 EU countries estimated that 3.6 million workers, 2% of the total workforce, are exposed to inhalable wood dust, with over half the workers exceeding 1 mg/m^3^ (Kauppinen et al. 2006). In Italian carpentries, the mean exposure was mostly above 1 mg/m^3^ inhalable wood dust, and only 3 of 16 workstations below this level (Gioffrè et al. 2012). Similarly, in the UK, median exposure in joineries and furniture manufacturer were above 1 mg/m^3^.

To our knowledge, this is the first report on resin acid exposure in the woodworking industry, whereas exposure in wood pelleting industry and sawmills have previously been published (Eriksson et al. 1997; Teschke et al. 1999; Hagström et al. 2008; Straumfors et al. 2018). The resin acid exposure (GM 1.12 µg/m^3^) were higher than the wood pelleting industry (GM 0.62 µg/m^3^), but lower than in Canadian (GM 8.04 µg/m^3^) and Norwegian (GM 7.5 µg/m^3^) sawmills.

Microbial exposure levels, including endotoxins (GM 1.4EU/m^3^), bacteria (GM 0.23×10^4^gc/m^3^), fungi (GM 0.06 ×10^4^gc/m^3^), and fungal spores (AM 2.2×10^4^/m^3^), were low, suggesting that microbial exposure is not a significant issue in the woodworking industry. These findings are consistent with earlier studies (Mandryk et al. 1999; Gioffrè et al. 2012), although a few potentially pathogenic bacteria have been reported. In contrast, African woodworkers had significantly higher exposures, with levels of GM 3.3 mg/m^3^ inhalable dust and 91 EU/m^3^, endotoxins in Tanzania (Rongo et al. 2004), and similar levels in Mozambique (Chamba et al. 2023).

The exposures to monoterpenes (GM 260 µg/m^3^), formaldehyde (GM 17 µg/m^3^) and acetaldehyde (GM 1 µg/m^3^) were well below the respective Norwegian OELs (140 mg/m^3^ for monoterpenes, 0.37 mg/m^3^ for formaldehyde and 45 mg/m^3^ for acetaldehyde (Norwegian Labour Inspection Authority 2024). These values were also lower than the 7.8 mg/m^3^ monoterpenes reported in the Danish furniture industry (Hagström et al. 2012) and the wood pelleting industry (GM 0.77 mg/m^3^ of α-pinene) (Hagström et al. 2008). Although the processes can be significantly different, both wood working and furniture industry use dried wood, whereas where fresh wood is cut or used, such as sawmills, monoterpene emissions can be higher (Straumfors et al. 2018).

### Impact of air recirculation

The use of air recirculation impacted exposures differently across company types. Workers producing building elements experienced higher wood dust exposure from softwood when air recirculation was used, whereas door and window manufacturers had significantly lower exposures. All companies working with a mixture of soft and hard wood always used air recirculation, preventing direct comparison with those not using it. Notably, 25% of the measurements exceeded the OEL for inhalable wood dust of 1 mg/m^3^.

Monoterpene concentrations were lower in companies using air recirculation, likely due to filters removing dust-bound volatile components. Although these filters are designed to capture dust rather than gases, they may help reduce VOCs like monoterpenes. This contrasts with findings from the Danish furniture industry, where air recirculation was associated with higher monoterpene exposures (Hagström et al. 2012). However, there are important differences between the two industries. Whereas woodworking primarily involves solid wood, which may have natural emissions of monoterpenes from the wood, and limited use of chemicals, the furniture industry involves more intensive use of VOC-emitting material like adhesives (formaldehyde-based resins), finishes and coatings (paints, varnishes, lacquers, and stains). The furniture industry may in addition be larger in scale, also contributing to higher levels.

Microbial exposures, particularly to bacteria, were higher in companies using air recirculation. One possible explanation for this observation may be that recycling creates growth conditions for bacteria by preserving heat and moisture. However, the microbial load was not large enough to suspect growth in the workshops.

### Impact of air humidification

Humidification systems are used to maintain optimal relative humidity in workplaces, balancing the moisture content of the wood products to preserve their quality. The humidification systems improved air quality by reducing inhalable wood dust exposure by 2.4 times when processing mixed wood. This reduction likely occurred as water droplets bound to dust particles, causing them to settle. However, this effect was observed only for inhalable dust, and humidification did not affect total dust levels from softwoods. This is likely because the inhalable dust fraction includes larger particles (up to 100 µm) that are more prone to absorb moisture and settle than small ones. The efficiency of humidification depends on droplet size, with smaller droplets being more effective at reducing inhalable dust, while additional filtration may be needed for finer particles (Hinds 1999). The dust-settling effect may also explain the tenfold reduction in inhalable resin acid exposure observed among workers manufacturing interior products at companies using air humidification (GM 344 ng/m^3^) compared to similar companies without air humidification (GM 3323 ng/m^3^). However, as humidification had no significant effect on resin acid exposure overall, company-specific factors likely play a role.

Interestingly, the monoterpene exposure was higher in companies with humidification systems, though the direct causal link remains unclear. Higher humidity may increase monoterpene volatility, raising airborne concentrations, as seen in sawmills (Straumfors et al. 2018). Additionally, humidification may alter air recirculation patterns or reduce ventilation efficiency, potentially concentrating monoterpenes (Hagström et al. 2012). Broader workplace conditions, such as machinery, layouts, and practices, could also contribute to elevated monoterpene levels. The fact that 99% of the monoterpene exposure variance could be explained by company or company type suggests that influential workplace factors were not fully captured. Notably, most monoterpene measurements above the detection limit were from one single company using humidification but not air recirculation.

Humidification systems also increased the exposure to bacteria, endotoxins, and, to a lesser extent, fungi. This may be because these systems provide the moisture microorganisms need to survive, while dry conditions cause desiccation. Additionally, microorganisms may have been introduced via the water and dispersed through the humidification process. While exposure levels remained below proposed thresholds, health effects depend not only on the quantity but also on the composition and pathogenicity of microorganisms (Berg et al. 2020). However, identifying species to assess health risks was beyond the scope of this study.

### Company types and company-specific exposure indicators

The potential exposure indicators were highly variable as they were tailored to individual companies, making it challenging to account for the variability in the data. The variability in exposure was best attributed to differences between companies, which reflected the diversity of work practices, products, and environmental conditions. Explained variances for total and inhalable wood dust were 18 and 21%, respectively, which were lower than the factory-based (28%) and factory- and machine-based (32%) models in the furniture industry (Schlünssen et al. 2008; Hagström et al. 2012). They were also lower than the task- and season-based models developed for small-scale wood industries in Tanzania (Rongo et al. 2004).

Several production-type and company-specific factors are known to impact wood dust exposure. Effective dust extraction systems have been proven to lower dust concentrations, while certain tasks and work processes, such as grinding, using compressed air, handheld tools, or fully automatic machines, as well as dry brushing and cleaning, are associated with higher wood dust exposure. Additionally, smaller workshops with fewer than 20 employees tend to have elevated exposure levels (Scheeper et al. 1995; Brosseau et al. 2001; Rongo et al. 2002; Schlünssen et al. 2008; Douwes et al. 2017). Companies employed various cleaning methods, alternating between compressed air, brooms, vacuuming, and wet cleaning. The most common cleaning method was compressed air, which was used to blow the workstation clean, while wet cleaning was rarely used. Compressed air and brushing generate significant airborne dust, while vacuuming and wet cleaning are more effective at dust removal (Schlünssen et al. 2008; Douwes et al. 2017). In British woodworking manufacturers, 91% used brushes, 95% used vacuum cleaning, and 41% used compressed air (Simpson et al. 2024). Frequent cleaning has been shown to reduce monoterpene exposure (Hagström et al. 2012), and alternatives such as vacuum extraction on handheld tools can reduce dust exposure by up to 30% (Douwes et al. 2017).

### Study limitations

A limitation of this study is that all inhalable wood dust measurements from companies working with mixed woods were taken with air recirculation. Future summer measurements, when air recirculation is less common, could improve comparisons but may be influenced by natural ventilation through open doors and windows. While a larger data set would provide more power to group-wise comparisons, the study’s strength lies in the substantial number of companies and repeated personal exposure measurements, making it representative of the woodworking industry in Norway and similar cold-climate countries.

## Conclusions

The use of air recirculation impacted the individual exposures at the various type of companies differently, reducing the monoterpene exposure, but increasing the microbial exposure. Air humidification reduced the inhalable dust exposure across company type but increased monoterpene and microbial exposure. Variability in exposure was primarily influenced by company-specific practices, product types, and workplace conditions. Preventive measures tailored to the individual companies are therefore recommended, and optimized ventilation, humidification systems, and effective cleaning routines should be considered to reduce exposure. Overall, while average exposure levels were low, significant variability indicates the necessity for targeted interventions to ensure all workers remain below the exposure limit.

## Supplementary material

Supplementary material is available at *Annals of Work Exposures and Health* online.

wxaf027_suppl_Supplementary_Tables_S1-S13

## Data Availability

The data underlying this article will be shared on reasonable request to the corresponding author.
