# Supplementary material for: Impact of air recirculation and humidification systems on wood dust exposure during woodworking
Source: Ann Work Expo Health. 2025 Jun 2;69(6):652–64. doi: 10.1093/annweh/wxaf027 (PMC12262046; doi:10.1093/annweh/wxaf027)
Supplement: wxaf027_suppl_Supplementary_Tables_S1-S13 [file wxaf027_suppl_supplementary_tables_s1-s13.pdf]

Supplementary tables to

Impact of air recirculation and humidification systems on wood dust exposure during woodworking

Anne Straumfors*, Ine Pedersen, Erika Zardin Brinchmann, Torunn Kringlen Ervik, Anani Afanou, Kristine H. Anmarkrud, Monica Eidhammer, Oda AH Foss, Nils Petter Skaugset

National Institute of Occupational Health, P.O. box 5330 Majorstuen, Oslo, Norway

*Correspondence: anne.straumfors@stami.no

Table S1. Flow rate (L/min) and sampling time (minutes) and samples air volume (m^3) for each sampler in the study

Sampler	Dust fraction/component	Flow rate	Time [mean (minimum-maximum)]	Volume [mean (minimum-maximum)]
CIS sampler	Inhalable dust	3.5	477 (140-544)	1.7 (0.5-2.1)
CIS sampler	Inhalable resin acids	3.5	476 (276-544)	1.7 (1.0-2.1)
Total dust cassette	Total dust	2	454 (62-740)	0.9 (0.4-1.5)
PAS-6 sampler	Inhalable endotoxin	2	470 (458-470)	0.9 (0.8-1.0)
Charcoal tubes	Volatile monoterpenes	0.05	471 (289-736)	0.02 (0.01-0.04)
Sep-Pak	Volatile aldehydes	0.05	482 (202-736)	0.02(0.01-0.04)

Table S2. Concentrations of individual resin acids and monoterpenes

Components	N	N<LOD	0-values	AM (SD)[1]	Median (min-max)	GM_{obs} (CI)[2]	GM_{adj} (CI)[3]
**Resin acids**							
Sum resin acids (μg/m³)	98	1	0	2.19 (2.95)	1.24 (0.11-19.9)	1.12 (0.88-1.43)	1.10 (0.82-1.46)
7-oxodehydroabietic acid (ng/m³)	98	33	6	90 (102)	54 (0.58-502)	50 (39-63)	48 (16-64)
Dehydroabietic acid (ng/m³)	98	1	1	873 (1003)	535 (21-6093)	491 (390-618)	478 (364-628)
Levopimaric acid (ng/m³)	98	58	35	58 (78)	23 (8.13-444)	36 (30-43)	36 (29-44)
Abietic acid (ng/m³)	98	8	7	692 (1208)	294 (18.52-9101)	269 (202-359)	261 (184-370)
Isopimaric acid (ng/m³)	98	10	7	479 (656)	227 (8.19-3923)	211 (160-279)	206 (147-289)
**Volatile components**							
Sum Monoterpenes (μg/m³)	77		13	6596 (14225)	79 (28-54095)	260 (149-458)	240 (115-501)
α-pinene (μg/m³)	77	30	14	3668 (7899)	48 (1-29204)	102 (54-192)	92 (40-211)
β-pinene (μg/m³)	77	52	30	279 (573)	18 (1 -2288)	35 (22-54)	34 (19-60)
D-limonen (μg/m³)	77	45	38	255 (538)	12 (1 -2084)	26(16-41)	25 (14-44)
3-karene (μg/m³)	77	52	29	2393 (5220)	15 (1-20519)	51 (27-97)	48 (21-109)
Formaldehyde (μg/m³)	97	3	0	29 (30)	19 (1-186)	17 (13-21)	17 (13-23)
Acetaldehyde (μg/m³)	97	7	0	2.0 (0.5)	1.8 (0.5-4.8)	1.9 (1.8-2.0)	1.9 (1.8-2.0)

Table S3. Correlation between exposure to individual resin acids and total and inhalable dust

. pwcorr logOXO logDHAA logLPA logAA logIPA logTotalstøv logInhalerbartstøv, sig star (0.05)

	logOXO	logDHAA	logLPA	logAA	logIPA
logOXO	1.0000				
logDHAA	0.8241*<br>0.0000	1.0000			
logLPA	0.5974*<br>0.0000	0.5763*<br>0.0000	1.0000		
logAA	0.7558*<br>0.0000	0.9540*<br>0.0000	0.5822*<br>0.0000	1.0000	
logIPA	0.7135*<br>0.0000	0.9405*<br>0.0000	0.4728*<br>0.0000	0.9711*<br>0.0000	1.0000
logTotaldust	0.3913*<br>0.0017	0.4120*<br>0.0009	0.1011<br>0.4345	0.4482*<br>0.0003	0.5149*<br>0.0000
logInhaledust	0.1931<br>0.2665	0.2606<br>0.1306	0.2828<br>0.0997	0.3372*<br>0.0476	0.2561<br>0.1376

Table S4. Mixed models of exposure components by product type

Model: $y = \mu + \beta_{product\ type} + u + \varepsilon$ (1)

	Random		Mixed		Effect	Estimated conc.
**Log Total dust**	**B**	**95%CI**	**B**	**95%CI**	**e^B**	**mg/m^3**
Constant	-1.23	-1.41;-1.05	-1,07	-1.29;-0.87	0.34	
Doors/windows			-0.58***	-0.93;-0.23	0.56	0.26
Interior products			0.33	-0.37-1.03	1.39	0.47
Building elements			Ref.		1	0.34
**Log Inhalable dust**	**B**	**95%CI**	**B**	**95%CI**	**e^B**	**mg/m^3**
Constant	-0.50	-0.81;-0.19	-0.67	-1.65-0.32	0.51	
Doors/windows			Ref.		1	0.51
Interior products			0.52	-0.54-1.59	1.69	0.70
Stairs			-0.21	-1.28-0.87	0.81	0.42
**Log Resin acid**	**B**	**95%CI**	**B**	**95%CI**	**e^B**	**ng/m^3**
Constant	7.00	6.71-7.29	6.78	6.32-7.24	884	
Doors/windows			1.04**	0.29-1.79	2.84	2512
Interior products			-0.16	-0.84-0.51	0.85	750
Stairs			0.33	-0.45-1.10	1.39	1226
Building elements			Ref.		1	884
**Log Endotoxins**	**B**	**95%CI**	**B**	**95%CI**	**e^B**	**EU/m^3**
Constant	0.38	-0.98-0.86	-0.88	-1.54;-0.22	0.42	
Doors/windows			2.41***	1.34-3.49	11.18	4.64
Interior products			2.23***	1.28-3.18	9.32	3.87
Stairs			0.58	-0.52-1.68	1.78	0.74
Building elements			Ref.		1	0.42
**Log Bacteria**	**B**	**95%CI**	**B**	**95%CI**	**e^B**	**gc/m^3**
Constant	8.13	7.66-8.60	7.09	6.48-7.70	1200	
Doors/windows			1.73***	0.90-2.56	5.64	6780
Interior products			0.92	-0.23-2.07	2.50	3010
Stairs			1.91**	0.70-3.13	6.78	8150
Building elements			Ref.		1	1200
**Log Fungi**	**B**	**95%CI**	**B**	**95%CI**	**e^B**	**gc/m^3**
Constant	6.46	6.06-6.86	6.18	5.51-6.85	480	
Doors/windows			0.53	-0.37-1.43	1.71	820
Interior products			-0.03	-1.31-1.24	0.97	470
Stairs			0.55	-0.79-1.89	1.98	830
Building elements			Ref.		1	480
**Log Monoterpenes**	**B**	**95%CI**	**B**	**95%CI**	**e^B**	**$\mu g/m^3$**
Constant	5.48	4.75-6.22	4.59	4.36-4.82		
Doors/windows			5.64***	5.22-6.05	280	27550
Interior products			-0.54**	-0.88;-0.20	0.58	57
Building elements			Ref.		1	98
**Log Formaldehyde**	**B**	**95%CI**	**B**	**95%CI**	**e^B**	**$\mu g/m^3$**
Constant	-4.07	-4.37;-3.78	-4.97	-5.19;-4.76	0.01	
Doors/windows			2.33***	1.98-2.68	10.3	0.07
Interior products			1.51***	1.19-1.83	4.52	0.03
Stairs			-0.06	-0.43-0.31	0.94	0.01
Building elements			Ref.		1	0.01

Log Acetaldehyde	B	95%CI	B	95%CI	e^B	µg/m³
Constant	-6.25	-6.29;-6.20	-6.17	-6.24;-6.10	0.002	
Doors/windows			-0.05**	-0.16-0.07	0.95	0.002
Interior products			-0.16*	-0.27;-0.06	0.85	0.002
Stairs			-0.13	-0.26;-0.01	0.87	0.002
Building elements			Ref.		1	0.002

*** p≤0.001; **p≤0.01; *p≤0.05

Table S5. Mixed models of exposure components by company

Model: $y = \mu + \beta_{company} + u + \varepsilon$ (2)

	Random		Mixed		
**Log Total dust**	**B**	**95%CI**	**B**	**95%CI**	**p**
Constant	-1.23	-1.41;-1.05	-0.473	-0.93;-0.02	0.042
AA			Ref.	-0.97-0.29	
C			-0.34	-1.83;-0.63	0.294
D			-1.23	-2.08;-0.94	0.000
E			-1.51	-1.37;-0.27	0.000
H			-0.82	-2.16;-0.29	0.003
J			-1.22	-1.02-0.28	0.010
M			-0.37	-2.64;-1.43	0.265
N			-2.03	-0.49-0.67	0.000
O			0.09	-1.24-0.05	0.764
P			-0.59	-1.04-0.73	0.072
Q			-0.15	-1.77;-0.35	0.732
S			-1.06	-2.58;-0.90	0.003
T			-1.74	-1.66;-0.16	0.000
U			-0.91	-0.21-1.20	0.017
V			0.50	-1.12-0.17	0.167
**Log Inhalable dust**	**B**	**95%CI**	**B**	**95%CI**	**p**
Constant	-0.50	-0.81;-0.19	-0.68	-1.12;-0.24	0.002
A			Ref		
B			0.74	0.13-1.35	0.018
G			-0.42	-1.30-0.46	0.349
I			0.02	-0.87-0.90	0.973
K			0.98	0.23-1.73	0.010
L			-0.50	-1.32-0.31	0.223
R			-1.39	-2.38;-0.40	0.006
**Log Resin acid**	**B**	**95%CI**	**B**	**95%CI**	**p**
Constant	7.00	6.71-7.29	7.11	6.63-7.59	0.000
A			Ref.		
B			-1.27	-1.95;-0.59	0.000
C			1.00	0.19-1.80	0.015
D			-0.64	-1.33-0.04	0.066
E			0.006	-0.69-0.71	0.986
F			0.72	0.06-1.38	0.034
**Log Endotoxins**	**B**	**95%CI**	**B**	**95%CI**	**p**
Constant	0.38	-0.10-0.86	-0.30	-0.91-0.31	0.332
A			Ref.		

B			1.34	0.48-2.21	0.002
C			2.30	1.28-3.32	0.000
D			1.11	0.20-2.02	0.017
E			-2.30	-3.19;-1.41	0.000
F			1.82	0.97-2.67	0.000
**Log Bacteria**	**B**	**95%CI**	**B**	**95%CI**	**p**
Constant	8.13	7.66-8.60	7.97	7.15-8.78	0.000
C			Ref.		
E			-0.18	-1.39-1.02	0.766
G			1.04	-0.15-2.23	0.087
I			2.07	0.88-3.26	0.001
J			-1.14	-2.15;-0.12	0.028
M			0.38	-0.59;1.36	0.438
**Log Fungi**	**B**	**95%CI**	**B**	**95%CI**	**p**
Constant	6.46	6.06-6.86	6.02	5,33-6,72	0.000
C			Ref.		
E			0.74	-0,24-1,72	0.139
G			0.70	-0,36-1,76	0.193
I			2.65	1,59-3,70	0.000
J			-0.03	-0,93-0,86	0.943
M			-0.02	-0,85-0,80	0.956
**Log Monoterpenes**	**B**	**95%CI**	**B**	**95%CI**	**p**
Constant	5.48	4.75-6.22	4.07	3.83-4.30	0.000
B			Ref.		
C			-0.06	-0.47-0.34	0.759
D			0.06	-0.27-0.40	0.719
E			1.04	0.69-1.39	0.000
F			6.16	5.81-6.51	0.000
**Log Formaldehyde**	**B**	**95%CI**	**B**	**95%CI**	**p**
Constant	-4.07	-4.37;-3.78	-5.03	-5.32;-4.75	0.000
A			Ref.		
B			1.68	1.28-2.08	0.000
C			1.36	0.89-1.83	0.000
D			-0.11	-0.51-0.30	0.613
E			0.24	-0.17-0.66	0.251
F			2.39	2.00-2.79	0.000
**Log Acetaldehyde**	**B**	**95%CI**	**B**	**95%CI**	**p**
Constant	-6.25	-6.29;-6.20	-6.30	-6.39;-6.21	0.000
A			Ref.		
B			-0.07	-0.19-0.06	0.312
C			0.04	-0.11-0.18	0.642
D			0.02	-0.11-0.15	0.793
E			0.26	0.13-0.39	0.000
F			0.08	-0.04-0.21	0.178

Table S6. Mixed models of total dust exposure by product type and air recirculation

Model: $y= \mu+\beta_{product\ type}+ \beta_{air\ recirculation}+ \beta_{product\ type,\ air\ recirculation} + u +\varepsilon$ (3)

	Random		Mixed		
**Log Total dust**	**B**	**95%CI**	**B**	**95%CI**	**p**
Constant	-1.23	-1.41;-1.05	-0.81	-1.37;-0.26	0.004
Building elements			Ref.		
Doors/windows			-1.09	-1.68;-0.50	0.000
Interior products			0.06	-0.79;0.91	0.888
Air recirculation			Ref.		
No air reciculation			-0.56	-1.17-0.05	0.073
Door/window#yes			Ref.		
Door/window#no			1.17	0.40-1.93	0.003

Table S7. Mixed model of inhalable dust exposure by air humidifiction

Model: $y= \mu+ \beta_{air\ humidification} + u +\varepsilon$ (4)

	Random		Mixed		
**Log Inhalable dust**	**B**	**95%CI**	**B**	**95%CI**	**p**
Constant	-0.50	-0.81;0.19	-0.58	-0.91;-0.25	0.001
Air humidification			Ref.		
No air humidification			0.89	0.02-1.75	0.044

Table S8. Mixed model of resin acid exposure by product and air humidification

Model: $y= \mu+ \beta_{product\ type}+ \beta_{air\ humidification}+ \beta_{product\ type,\ air\ humidification} + u +\varepsilon$ (5)

	Random		Mixed		
**Log Resin acids**	**B**	**95%CI**	**B**	**95%CI**	**p**
Constant	7.00	6.71-7.29	4.51	3.61-5.42	0.000
Building elements			Ref.		
Doors/windows			3.32	2.29-4.34	0.000
Interior products			1.33	0.57-2.09	0.001
Stairs			2.60	1.56-3.63	0.000
Air humidification			Ref.		
No air humidification			2.27	1.44-3.10	0.000

Table S9. Variance partition coefficients and variances explained by mixed models (% explained variance)

	Variance partition component (%)		Explained variance (%)
	σ^2BW	σ^2WW	σ^2TOT
**Model 1. Product**			
Total dust	48	52	3
Inhalable dust	58	42	7
Resin acids	48	52	9
Endotoxins	51	49	53
Bacteria	46	54	29
Fungi	34	66	3
Monoterpenes	52	48	99
Formaldehyde	47	52	43
Acetaldehyde	49	51	0,2
**Model 2. Companies**			
Total dust	39	61	18
Inhalable dust	51	49	21
Resin acids	38	62	23
Endotoxins	31	69	69
Bacteria	40	60	37
Fungi	27	73	27
Monoterpenes	50	50	99
Formaldehyde	47	53	43
Acetaldehyde	49	51	1
**Model 3. Product#air recirculation**			
Total dust	46	54	5
**Model 4. Air humidification**			
Inhalable dust	59	41	4
**Model 5. Product#air humidification**			
Resin acids	40	60	22

σ^2BW: % variance explained by differences between workers: (σ^2bw/ σ^2tot)*100%; σ^2WW: % variance explained by differences within workers (σ^2ww/ σ^2tot)*100%; σ^2TOT: % total variance explained by the mixed effects of the model (($\sigma^2 tot_{random}$- $\sigma^2 tot_{mixed}$)/ $\sigma^2 tot_{random}$) × 100%.

Tables S10-S13. The number of samples distributed over exposure relevant company variables.

Table S10. Air recirculation, air humidification and wood type

	Companies	Samples	Air recirculation		Air humidification		Wood type	
**Company type**	**Number**	**Number**	**Yes**	**No**	**Yes**	**No**	**Soft**	**Mixture of Hard and soft**
Building elements	11	148	22	108	0	114	149	0
Door/Window	5	82	52	30	76	0	76	6
Interior products	4	51	51	0	23	28	18	33
Stairs	3	42	42	0	36	0	0	42

Table S11. Ventilation and dust extraction

	Companies	Samples	Ventilation operating time after work[1]			Individual dust extraction at workstation		
**Company type**	**Number**	**Number**	**Off**	**On**	**Reduced**	**Yes**	**No**	**Some**
Building elements	11	148	42	37	0	96	6	12
Door/Window	5	82	55	0	21	82	0	0
Interior products	4	51	41	0	10	41	0	10
Stairs	3	42	42	0	0	42	0	0

Table S12. Dust filter and air supply filter

	Companies	Samples	Coarse filter/dust extraction				Dust filter/air supply		
**Company type**	**Number**	**Number**	**Yes**	**Channel filter[1]**	**No**	**Bagfilter[2]**	**Channel filter[1]**	**No**	**Bagfilter[2]**
Building elements	11	148	18	0	6	37	0	30	31
Door/Window	5	82	30	21	6	25	21	19	6
Interior products	4	51	0	0	18	33	0	10	41
Stairs	3	42	0	6	0	36	6	18	18

Table S13. Cleaning type and frequency

	Compressed air/blowing	Broom	Vacuum cleaner	Wet sway/washing
Several times a day	111	39	20	12
Daily	90	54	70	0
2-3 times a week	18	11	0	0
Weekly	6	96	66	18
Rarer/if needed	48	41	18	24
Not used	4	52	49	222
Don't know/not stated	47	31	31	47
